# Hand Grip Strength as an Index of Health Does Not Associate With Insomnia: A Cross-Sectional Study

**DOI:** 10.7759/cureus.26170

**Published:** 2022-06-21

**Authors:** Abdullah O Alrasheed, Ahmad M Samman, Omar E Tarabzoni, Mohammed S Alnumani, Mohammed Alkhamis, Ahmed Hilabi, Feras S Alharbi, Abdulrahman S Alraddadi, Awad Almuklass

**Affiliations:** 1 College of Medicine, King Saud Bin Abdulaziz University for Health Sciences, Riyadh, SAU; 2 Basic Medical Sciences, King Saud Bin Abdulaziz University for Health Sciences, Riyadh, SAU; 3 Department of Research Office, King Abdullah International Medical Research Center, Riyadh, SAU

**Keywords:** prevention, health, sleep, handgrip strength, insomnia

## Abstract

Introduction

Insomnia is one of the most prevalent diseases globally, with many adults around the world suffering from at least one of its symptoms. It has a significant effect on the body's normal physiology and may lead to the development of chronic diseases that impair the main functional domains of health and cognition if left untreated. Handgrip strength (HGS) has previously been linked to several diseases that occur in tandem with insomnia. Thus, this study aimed to investigate the association between HGS and insomnia.

Materials and methods

This is a cross-sectional study in which the involved participants were approached in different locations. The participants were surveyed using the Insomnia Severity Index (ISI) to evaluate the presence of insomnia. A hydraulic hand dynamometer was used to measure the HGS of the participants' right and left hands.

Results

A total of 494 questionnaires were collected, including 365 (74%) males and 129 females (26%). About 16% of females had insomnia, compared to 15% of males. There was no significant association between gender and insomnia (P=0.873). The difference between the mean HGS for the right and left hands among males and females was not statistically significant (P>0.05). The correlation coefficients of the right and left HGS scores with insomnia were r=0.019 and r=0.018, respectively, which showed no statistically significant association (P>0.05).

Conclusions

The study found no significant association between HGS and insomnia. The study recommends conducting further large-scale studies focusing on specific groups in the population to understand the relationship between HGS and chronic sleep disturbance.

## Introduction

Insomnia is a sleeping disorder in which people have difficulties falling asleep, staying asleep, and/or awakening early without the ability to return to sleep [1]. In some sleep literature, the presence of polysomnographic evidence of low-quality sleep is used to define insomnia. Therefore, the presence of frequent nocturnal awakenings, a long sleep latency, prolonged periods of wakefulness during the sleep period, and/or even recurring transient arousals are taken as evidence of insomnia [2]. The prevalence of insomnia can be estimated based on two main factors: the criteria used to define insomnia and, more significantly, the population studied [3]. For example, population-based studies have developed a consensus that approximately 30% of adult samples from different countries claim one or more symptoms of insomnia [4]. Moreover, the application of the Diagnostic and Statistical Manual of Mental Disorders, Fourth Edition (DSM-IV) includes the additional requirements where insomnia symptoms must persist for at least one month and do not entirely occur in the presence of another sleep disorder, mental disorder, or the direct physiological effects of a substance or medical condition, which yields a prevalence of approximately 6% [5].

Insomnia may increase the risk of developing multiple types of diseases that range from psychological domains such as anxiety and depression to the biological domain such as high blood pressure, coronary artery disease, diabetes, and even cancer due to the distribution of slow-wave sleep (SWS) or deep sleep, where the most healing physiological mechanisms take place [6]. These healing mechanisms include reducing heart rate, blood pressure, sympathetic nervous system activity, an increase in vagal tone, the inhibition of the hormones of the hypothalamic-pituitary-adrenocortical (HPA) system, and an increase in growth hormone (GH) and prolactin. Laboratory studies showed that sleep deprivation can adversely affect these metabolic hormones, which significantly affect skeletal muscle growth and function [7-9].

Handgrip strength (HGS) reflects the strength of upper skeletal muscles and their functional integrity that can be measured with what is known clinically as the Jamar grip dynamometer, which has well-grounded indices for reliability and validity [10]. Handgrip strength as a health biomarker is often used to document the development of muscle strength during rehabilitation programs or even after hand surgery as a measure that indicates the level of hand function. It is more useful when multiple values are taken over time to track the progression or regression of physical performance or to compare the result to a normative reference, which many peer-reviewed studies provide.

Given that HGS has been found to have an inverse association with metabolically unhealthy populations and is linked to many diseases that co-exist with insomnia, implementation of handgrip strength as a routine for screening could be a crucial preventative measure to avoid an impending health crisis [11,12]. Thus, this study aims to identify any possible association between insomnia and HGS in the general population.

## Materials and methods

Setting and participants

The represented cross-sectional study was ethically approved by the IRB at MNGHA, Riyadh, Saudi Arabia. It was carried out in Riyadh to assess the relationship between HGS and insomniac symptoms among the general population. Data collection took place in various public areas such as malls, hospitals, parks, and scientific gatherings to target the general population with no preference. After participants' approval and consent, they were given a self-administered questionnaire to fill out, and then a hand dynamometer was used to measure the handgrip strength. For sample size calculation and estimation, a cyber calculator was used, and the margin of error was set at 5%, with a confidence level of 95%, and the population of Riyadh is 7.7 million, which results in an acceptable minimum sample size of 384. The data were collected from 505 participants, including males and females from various age groups. However, some data were lost during the research process, and the statistical analysis was performed on 494 participants instead.

Questionnaire

Investigators approached and educated the participant on the research parameters and then had the participant fill out a questionnaire that inquired about the demographic data and screened for insomnia using the Insomnia Severity Index (ISI), which in correlation with sleep diaries and polysomnography, scored 86.1% sensitivity and 87.7% specificity for detecting insomnia cases in multiple study samples [13]. Multiple studies have shown that the ISI has adequate internal consistency and is a reliable self-report measure to evaluate sleeping difficulties [14]. The questionnaire uses a Likert scale where respondents rate each element from 0 to 4. The final score is then interpreted based on the given guidelines in Table 1.

**Table 1:** Guidelines for scoring/interpretation.

Outcome	Reference range
No clinically significant insomnia	0-7
Subthreshold insomnia	8-14
Clinical insomnia	<15

It includes seven questions that examine the nature and symptoms of sleeping difficulties and, overall, the level of distress caused by sleeping problems. The questionnaire also included a brief medical history regarding chronic diseases, diseases influencing grip strength performance, and general physical examinations, including height and body weight measurements.

Handgrip measurements

Investigators used the LAFAYETTE Hydraulic Hand Dynamometer, model J0010538 (Lafayette Instruments, Lafayette, IN), to assess both hands' grip strength. For an accurate and reproducible outcome, the hand dynamometer automatically retains the highest reading on a special peak-hold needle, which remains on the gauge until the examiner resets it. Optimization and sequence of steps were established through several steps to ensure standardization and quality of measurements. Grip strength was measured in a seated position with the elbow flexed at 90°. The dynamometer's gauge was set at zero before each trial. Participants got two trials for each hand. The procedure was explained to each participant thoroughly from beginning to end by the investigators. During testing, the participant was strongly encouraged to exhibit the best possible force. The participant began by gradually increasing the strength during the procedure for three seconds and then maintained maximum strength for three seconds. The dynamometer output was measured in kilograms.

Statistical analysis

Data were entered and organized using Microsoft Excel (Microsoft® Corp., Redmond, WA) and then analyzed by an independent biostatistician using IBM statistical software SPSS (version-27, IBM SPSS, Armonk, NY). A chi-squared test was used to identify the association between gender and insomnia. An independent t-test was used to determine whether there was a difference between males and females in HGS. The one-way analysis of variance (ANOVA) was used to determine whether there is an association between HGS and insomnia. Multivariate logistic regression analysis was utilized to identify if age and gender would impact the association between the HGS and insomnia. Missing data were handled by eliminating any missing values through innate programming of IBM SPSS Statistics 25.0 (IBM, Armonk, NY) during analysis, and all statistical tests were regarded significant at P<0.05. In addition, due to some missing information regarding some categories in Tables 2-3, the total number of participants accounted for less than the total studied population. For ensuring consistency in reported data, the statistical discrepancy in the number of populations in males and females was explained in the legends of the respective tables.

**Table 2:** Chi-square test: the association between gender and insomnia. *Participants who failed to fill either the insomnia questionnaire or the gender question was not counted in this table. Look at the material and method section (Data analysis) for further clarification.

Variables			Insomnia scale				
			No clinical insomnia	Subthreshold	Clinical insomnia	Total	P-value
Gender	Male	Count	145	156	54	*355	0.873
		% Within gender	40.8%	43.9%	15.2%	100.0%	
	Female	Count	54	52	20	*126	
		% Within gender	42.9%	41.3%	15.9%	100.0%	
Total		Count	199	208	74	*481	
		% Within gender	41.4%	43.2%	15.4%	100.0%	

**Table 3:** One-way ANOVA: association between handgrip strength and insomnia. CI: confidence interval; SD: standard deviation. *Participants who failed to have their grip strength measured or did not fill the gender question were not counted in this table.

Gender	Performance	Insomnia scale	N	Mean (kg)	SD	95% CI		
						Lower	Upper	P-value
Male	Right hand average	No clinical insomnia	144	38.9	8.5	37.5	40.3	0.848
		Subthreshold	156	38.4	8.9	37	39.8	
		Clinical insomnia	54	38.9	8	36.7	41.1	
		Total	*354	38.7	8.6	37.8	39.6	
	Left hand average	No clinical insomnia	144	37.4	8.3	36.1	38.8	0.235
		Subthreshold	155	37	8.7	35.6	38.4	
		Clinical insomnia	54	39.3	8.4	37	41.6	
		Total	*353	37.5	8.5	36.6	38.4	
Female	Right hand average	No clinical insomnia	54	21.7	5.4	20.2	23.1	0.579
		Subthreshold	52	20.7	5.7	19.1	22.3	
		Clinical insomnia	20	20.5	5.2	18	23	
		Total	*126	21.1	5.5	20.1	22.1	
	Left hand average	No clinical insomnia	54	21	6.1	19.4	22.7	0.267
		Subthreshold	52	19.3	5.1	17.9	20.8	
		Clinical insomnia	20	19.8	4.6	17.6	21.9	
		Total	*126	20.1	5.5	19.2	21.1	

## Results

Four hundred and ninety-four participants were included in the study; the majority (N=365) were men. The mass number of the participants fall into the age category between 18 and 25 years old (N=252, 51%), and the remaining subjects were grouped into the following age categories: 169 (35%) were between the ages of 26 and 40, 38 (8%) were between the ages of 41 and 50, 30 (6%) were between the ages of 51 and 60, and 5 (1%) were over >60. The mean strengths of the right-hand and left-hand trials were 36.33 kg and 32.93 kg, respectively. Moreover, the mean handgrip strength of the right hand was 38.6 ± 8.6 kg and 21 ± 5.5 kg in males and females, respectively, while the mean for the left hand was 37.5 ±8.5 kg and 20.1 ± 5.5 kg for the same (Table 4). Out of the included population, 199 participants were classified as having "no clinical insomnia," 208 participants as having "subthreshold insomnia," and 74 participants as having clinical insomnia (Table 2).

**Table 4:** Independent t-test: comparison between males and females in handgrip strength. SD: standard deviation.

Variables		N	Mean (kg)	SD	P-value
Side	Gender				
Right hand	Male	365	38.6	8.6	<0.001
	Female	129	21.0	5.5	
Left hand	Male	365	37.5	8.5	<0.001
	Female	129	20.1	5.5	

Coefficients’ relationship

The independent t-test indicates a statistically significant difference in handgrip strength between genders (Table 3). The chi-square test showed no statistical significance in insomnia classification between males and females (Table 4). One-way ANOVA showed no statistically significant result between handgrip strength and insomnia classification (Table 2). Even when a multivariate regression analysis test was used to investigate the impact of age and gender on the relationship between handgrip strength and insomnia classification, the relationship remained statistically insignificant (Table 5).

**Table 5:** multivariate regression analysis test: the effect of age, gender, and insomnia on the dependent variables (right hand average). CI: confidence interval.

Variables		B	t	P-value	95% CI	
					Lower	Upper
Age category	18–25 years old	7.9	2.5	0.015	1.6	14.2
	26–40 years old	10.7	3.3	0.001	4.3	17.1
	41–50 years old	9.0	2.6	0.009	2.2	15.7
	51–60 years old	8.3	2.3	0.021	1.3	15.3
	>60 years old (ref)	0	Reference			
Gender	Male	17.5	21.6	<0.001	15.9	19.1
	Female (ref)	0	Reference			
Insomnia's classifications	Clinically insignificant insomnia	0.2	0.2	0.819	−1.9	2.3
	Sub-threshold insomnia	−0.2	−0.2	0.836	−2.3	1.8
	Clinically significant insomnia (ref)	0	Reference			

## Discussion

Numerous studies have investigated a wide range of variables relating to insomnia [5,8,12]; however, only one paper studied the relationship between handgrip strength and insomnia among old ladies [12]. To the best of our knowledge, this is the second study that attempted to analyze the association between insomnia and HGS. Other studies linked HGS to functional capacity in people older than 60 years, with cognition in the general population, with depression in a population between 40 and 79 years, and with psychological and social health in individuals who are over 85 years of age. Notably, these studies did not consider the general population in a cross-sectional manner as the present did; instead, they solely focused on the elderly [15-17].

Therefore, this study aimed to exclusively analyze the relationship between HGS and sleep among the general population. However, the study's findings did not show any significant association even when multiple indices such as age and gender were added to the equation. The deleterious effect of insomnia is much more likely to deteriorate the health of the elderly at a faster rate due to the age-related sleep changes resulting in early and fragmented sleep [18,19]. Furthermore, these pernicious sleep deprivation effects will manifest themselves as neurological, muscular, and psychological symptoms. The study's lack of significance might be attributed to the fact that much of our studied population is those who fell between the ages of 18 and 25, which is the least susceptible.

The previous study results that connected HGS to insomnia show that those who scored high HGS values have higher sleep satisfaction scores, while those who scored low HGS values have a higher score for insomnia with a significant result (P=0.032) [12]. This shows a direct association between HGS and sleeps data; nevertheless, the authors of the study have not contrasted with previous studies because, to the best of their knowledge, this association has not been previously analyzed [12]. Additionally, it is worth noting that the current study used the ISI to evaluate the presence of insomnia and focused on the general population. In contrast, the previous study used the Oviedo sleep questionnaire, a diagnostic aid questionnaire for insomnia and hypersomnia sleep disorders, and they focused on women who are over 65 years of age [12].

The limitation that we faced in this study is that we initially intended to collect a larger sample size, especially older adults; however, the COVID-19 situation sabotaged that goal. This study's strength is that it is the first paper that highlights the association between handgrip strength and insomnia among the general population, and it establishes a normative database for future research.

Recommendations

Due to the coronavirus disease 2019 (COVID-19) pandemic, data collection was restricted and did not meet the optimal target, especially for older adults. Most of the participants fall into the age category between 18 and 25 years old (N=257, 51%), while insomnia is more common at older ages (22). We suggest doing more studies with larger sample sizes and older ages to assess if there is any association between insomnia and HGS.

## Conclusions

In conclusion, this paper examined handgrip strength along with insomnia and whether they have an impact on each other. Handgrip strength was analyzed based on right and left average, and insomnia was classified based on its clinical symptoms. Moreover, these variables were subdivided even further based on the age category and gender to acknowledge the biological differences in these groups. However, the study found no significant association between HGS and insomnia. Therefore, we recommend further larger-size cross-sectional studies that focus on the local population to reflect the scale natively.
